# Impact of ablation ratio on 5-year postoperative posterior corneal stability after refractive surgery: SMILE and FS-LASIK

**DOI:** 10.1186/s40662-020-00218-y

**Published:** 2020-11-10

**Authors:** Meiyan Li, Danjuan Yang, Yu Zhao, Weiming Yang, Jianmin Shang, Xueyi Zhou, Peijun Yao, Dong Yang, Xue Lin, Xingtao Zhou

**Affiliations:** 1 Department of Ophthalmology, EYE & ENT Hospital, Fudan University, Shanghai, China; 2 NHC Key Laboratory of Myopia (Fudan University), Shanghai, China; 3 Shanghai Research Center of Ophthalmology and Optometry, Shanghai, China; 4 Department of Ophthalmology and Optometry, Children’s Hospital of Fudan University, Shanghai, China; 5 Department of Ophthalmology, Dalian Municipal Women and Children’s Medical Center, Dalian, China

**Keywords:** Posterior corneal elevation, Ablation ratio, SMILE, FS-LASIK, Myopia

## Abstract

**Background:**

To investigate the impact of the ablation ratio on 5-year postoperative posterior corneal stability in myopic eyes after small incision lenticule extraction (SMILE) and femtosecond laser-assisted in situ keratomileusis (FS-LASIK) surgery.

**Methods:**

A prospective, nonrandomized, cohort study: 80 eyes of 43 patients underwent SMILE surgery and 63 eyes of 32 patients underwent FS-LASIK surgery at the EYE & ENT Hospital, Fudan University. Ablation ratio was defined as lenticule thickness (SMILE cases) or ablation depth (FS-LASIK cases) divided by central corneal thickness (CCT). Posterior corneal elevation changes were recorded as posterior central elevation (PCE), posterior corneal surface at thinnest point (PTE) and posterior corneal mean elevation (PME). Patients were followed up at 6-month and 5-year interval to investigate the impact of the ablation ratio on posterior corneal elevation after SMILE and FS-LASIK surgery.

**Results:**

PCE dropped at the 6-month follow-up for both SMILE (decreased by −1.11 ± 2.93 μm, *P* < 0.05) and FS-LASIK groups (decreased by −0.46 ± 3.72 μm, *P* < 0.05). PTE also dropped in SMILE (reduced by −2.04 ± 3.02 μm, *P* < 0.05) and FS-LASIK group (reduced by −1.28 ± 4.21 μm, *P* < 0.05) at the 6-month follow-up. Stable PCE (elevation change: SMILE −0.28 ± 4.03 μm; FS-LASIK 0.79 ± 4.13 μm, *P* > 0.05) and PTE (elevation change: SMILE −0.08 ± 4.28 μm; FS-LASIK 1.42 ± 3.85 μm, *P* > 0.05) for both groups were recorded at the 5-year follow-up compared to the 6-month visit. Ablation ratio was strongly correlated with 5-year postoperative PCE (β = 2.68 ± 1.05, *P* < 0.01) and PTE (β = 2.35 ± 1.17, *P* < 0.05). Cut-off value for 5-year postoperative raised PCE and PTE was 27.3 and 27.1%, respectively.

**Conclusions:**

Ablation ratio was strongly correlated with postoperative posterior corneal elevation in a 5-year follow-up in both SMILE and FS-LASIK groups. PCE and PTE underwent slight backward displacement 6-month postoperatively and remain stable at the 5-year follow-up. Threshold of the ablation ratio for resisting forward displacement of posterior corneal surface was 27.3 and 27.1% for SMILE and FS-LASIK groups, respectively.

## Background

Small incision lenticule extraction (SMILE) surgery is a flap-free, small-incision refractive operation introduced in 2011 by Sekundo and Shah in correcting myopia with stable visual outcomes in both the short- and long-term and is believed with better post-operative corneal stability compared with femtosecond laser-assisted in situ keratomileusis (FS-LASIK) [1–4]. To date, about 3 million SMILE surgeries have been carried out worldwide in more than 70 countries [5]. Iatrogenic corneal ectasia is one of the most feared complications following nearly all keratorefractive surgeries in that it disturbs the integrity of the cornea. Keratoectasia following SMILE surgeries is rare. Only 7 eyes of four patients have been reported in literature [6].

Posterior corneal elevation is neither affected by surgery incision or flap creation nor easily influenced by tear film, and thus was reported as an early indicator for evaluating postoperative corneal stability with good repeatability and reliability [7–9]. Previous studies reported that risk factors of ectasia post LASIK and photorefractive keratectomy (PRK) surgeries include residual bed thickness (RBT), preoperative corneal thickness, abnormal corneal topography, age and high myopia state [10]. However, risk factors of forward posterior corneal displacement post SMILE and FS-LASIK surgery in the long term remain unknown.

This study aims to record a 5-year follow-up of posterior corneal elevation change and reveal the correlation between an individualized metric “ablation ratio” and postoperative posterior corneal stability.

## Methods

### Study design and participants

Eighty eyes of 43 patients undergoing SMILE surgery and 63 eyes of 32 patients undergoing FS-LASIK surgery at Eye and ENT Hospital of Fudan University were enrolled in this prospective, nonrandomized, cohort study from December 2011 to March 2012 and from January to February 2013. The eligibility criteria for both surgical groups were: age > 18 years, corrected distance visual acuity (CDVA) ≥ 20/20, refractive error remained stable in recent 2 years without other ocular pathologies, calculated RBT without epithelium > 280 μm. Patients were routinely examined and met the criteria for either SMILE or FS-LASIK. ﻿Patients with systemic or other ocular diseases, history of ocular surgeries or trauma, pregnant or breast-feeding state were excluded from this study. The exclusion criteria for both surgical groups were the same. This study was approved by Ethical Committee of Eye and ENT Hospital of Fudan University Review Board (KJ2010–18) and the study was conducted in accordance with the tenets of the Declaration of Helsinki. Each patient chose one of the two procedures after fully understanding the risks and benefits of both surgeries and provided written informed consent. Detailed patient characteristics were summarized in Table 1.

**Table 1:** Patient characteristics

	SMILE group	FS-LASIK group	***P*** value
**Pre-operative data**			
Patient number	43	32	N/A
Study eye number	80 (38 left, 42 right)	63 (32 left, 31 right)	N/A
Sex (Male/Female) ^c^	14/29	5/27	0.1
Age (years) ^b^	30 (25 ~ 32)	27 (23 ~ 32)	0.13
Spherical refractive error (D) ^a^	−5.98 ± 1.52	−7.70 ± 2.28	< 0.01
Cylindrical refractive error (D) ^b^	−0.50 (−1.00 ~ −0.25)	−1.00 (−1.50 ~ −0.75)	< 0.001
Spherical equivalent (D) ^a^	−6.33 ± 1.56	−8.26 ± 2.39	< 0.001
CDVA (logMAR) ^a^	−0.03 ± 0.05	−0.02 ± 0.04	< 0.01
Axial length (mm) ^b^	26.05 (25.48 ~ 26.76)	26.24 (25.50 ~ 27.25)	0.38
Central corneal thickness (μm) ^b^	561 (527 ~ 573)	545 (526 ~ 568)	0.28
**Intra-operative data**			
Cap/Flap diameter (mm)	7.5	8	N/A
Cap/Flap thickness (μm)	100–110	100	N/A
Lenticule thickness (SMILE, μm) or Ablation depth (FS-LASIK, μm) ^b^	132 (104 ~ 144)	142 (128 ~ 158)	< 0.001

*N/A=* not applicable; *CDVA*=corrected distance visual acuity; *logMAR*=logarithm of the minimal angle of resolution; *UDVA*=uncorrected distance visual acuity; *D*=diopter; *SMILE*=small incision lenticule extraction; *FS-LASIK*=femtosecond laser-assisted in situ keratomileusis. Data are mean ± standard deviation or median and interquartile range for data that were normally or non-normally distributed. *P* values less than 0.05 were considered statistically significant.

^a^ Student’s t-test

^b^ Wilcoxon rank-sum test

^c^ Chi-squared test

### Data collection and analysis

Elevation of posterior corneal surface, corneal thickness and topography were measured by Pentacam HR (Oculus GmbH, Wetzlar, Germany). One picture of each enrolled eye was taken in the preoperative examination and each follow-up. An image with “OK” statements under inspection window and central 9 mm corneal area covered qualified for further analysis. Posterior corneal elevation changes were recorded in three parameters: posterior central elevation (PCE), posterior corneal surface at thinnest point (PTE) and posterior corneal mean elevation (PME). PME was the average height of 8 points taken from a 4 mm diameter concentric circle (i.e., 2 mm from the center at 0°, 45°, 90°, 135°, 180°, 225°, 270° and 315° semi-meridians). Reference best fit sphere (BFS) was determined by the central 8 mm area of the preoperative cornea to ensure that the BFS of all postoperative maps were comparable with preoperative data. Ablation ratio was calculated as lenticule thickness/central corneal thickness (CCT) in SMILE group and ablation depth/CCT in FS-LASIK group. Lenticule thickness and ablation depth were extracted from each surgical laser system.

### Surgical procedures

SMILE surgery was performed by VisuMax femtosecond laser system (Carl Zeiss Meditec AG, Jena, Germany) with laser settings of 500 kHz repetition, 130 nJ pulse energy. A spot distance of 2.5 μm was used for the lenticule cut and cap cut and 2.0 μm for the lenticule side-cut and cap side-cut. An S-size contact glass was applied. The attempted treatment center was the corneal vertex. The corneal cap thickness was 100 to 110 μm. Target lenticule diameter (optical zone) was set to 6.0–6.8 mm depending on preoperative corneal thickness and refractive error to be corrected. Tissue arcade diameter was set 1 mm larger than the diameter of the lenticule. The lenticule side cut and opening incision side cut were fixed at 90 degrees with a circumferential width of 2.0 to 4.5 mm.

High-precision flap in FS-LASIK surgery was created by the same VisuMax femtosecond laser system with a pulse energy of 185 nJ. Stromal ablation was performed by MEL 80 excimer laser (Carl Zeiss Meditec, Oberkochen, Germany) with laser settings of 250 Hz repetition and 1 mJ pulse energy. The intended flap thickness was 100 μm. The hinges were set at a superior orientation with a hinge length of 4.0 mm. Patients wore bandage soft contact lenses (ACUVE OASYS, Inc., FL, USA) for 1 day after surgery. S cone was used in both procedures. All surgeries were conducted by an experienced refractive surgeon (ZXT).

Postoperative topical medication regimens were identical for both groups consisting of an ophthalmic solution of levofloxacin ﻿(Cravit®; Santen, Osaka, Japan) four times per day for 7 days, a 0.1% fluorometholone solution (Flumetholon®; Santen, Osaka, Japan) from 8 times to 1 time per day over a course of 24 days and a tear supplement 4 times per day for 1 month.

### Follow-up and statistical analysis

Patients were followed up at 6-month and 5-year interval with postoperative visual outcomes and posterior corneal elevation data collected for evaluation. Categorical variables were summarized by frequencies and percentages and tested by Chi-squared test. Continuous variables with a normal distribution were summarized by mean ± standard deviation and tested by t-test. Continuous variables with a nonnormal distribution were summarized by median and interquartile range, tested by Wilcoxon rank-sum test. A sample size of 51 eyes per group was calculated to detect a 1.4 μm difference of posterior corneal elevation between two surgical groups with an intended power of 90% and a significance level of 5% [11, 12]. Mixed effect model was applied to analyze postoperative posterior corneal elevation change with adjustment for preoperative spherical equivalent. Univariable and multivariable analysis were performed to determine independent relationship between baseline parameters and potential risk factors of postoperative posterior corneal elevation measurements. The analysis was performed with generalized estimating equations (GEE). To avoid the impact of the fellow eye in this study, we applied GEE with the fellow eye as covariate, namely eyes (two per individual) were clustered at subject level. Correlation coefficient β indicates the magnitude and direction of posterior corneal elevation change with per unit increase of the risk factor parameter. The cut-off value of ablation ratio was determined by the point of maximum Youden’s index (*J*). *J* can be formally defined as *J* = max_*c*_ {Se (*c*) + Sp (*c*) − 1}, in which Se (*c*) and Sp (*c*) were sensitivity and specificity at a certain point *c* [13]*.* Statistical analyses were performed with SPSS (version 22.0; IBM Corp., Armonk, NY). *P* < 0.05 was considered statistically significant.

## Results

### Visual outcomes

**Fig. 1:**
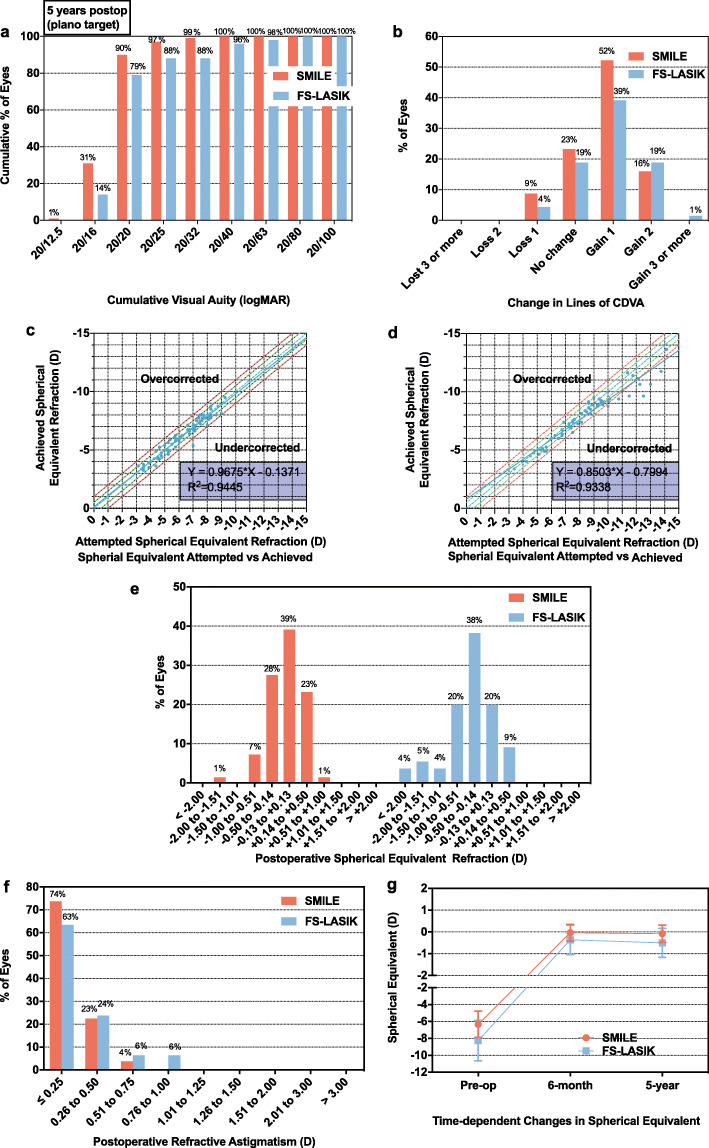
Visual outcomes of SMILE and FS-LASIK groups. **a**. Five-year postoperative cumulative uncorrected distance visual acuity; **b**. Five-year postoperative corrected distance visual acuity; **c**. Attempted versus achieved spherical equivalent refraction after SMILE at five-year follow-up; **d**. Attempted versus achieved spherical equivalent refraction after FS-LASIK at five-year follow-up; **e**. Five-year postoperative spherical equivalent refraction change; **f**. Five-year postoperative refractive astigmatism; **g**. Time-dependent changes of spherical equivalent. SMILE = small incision lenticule extraction; FS-LASIK = femtosecond laser-assisted in situ keratomileusis; CDVA = corrected distance visual acuity; logMAR = logarithm of the minimal angle of resolution

Forty-three patients (80 eyes) undergoing SMILE surgery and 32 patients (63 eyes) undergoing FS-LASIK surgery were included in this study. Thirteen patients (23 eyes) were lost in the 6-month follow-up but reengaged in the 5-year follow-up. Nine (12%) patients in total (17 eyes, SMILE group, 11 eyes, FS-LASIK group: 6 eyes) were lost in the 5-year follow-up because they were untraceable via phone numbers. No patients encountered post-operative keratectasia, dry eye, infectious keratitis, and other complications in this study. In the five-year follow-up of both groups, no eyes lost 2 lines or more of CDVA postoperatively. 90% (62/69) eyes in the SMILE group and 79% (45/57) eyes in the FS-LASIK group achieved uncorrected distance visual acuity (UDVA) of 20/20 or better. Safety and efficacy indices were 1.18 and 1.14 for the SMILE group, 1.21 and 1.06 for the FS-LASIK group. In the 5-year follow-up, spherical equivalent of 90% (62/69) eyes of the SMILE group were within 0.5 D of target correction and 99% (68/69) eyes within 1.0 D of target correction. 96% (66/69) eyes in the SMILE group had astigmatism ≤ 0.5 D. Spherical equivalent of 70% (39/57) eyes in FS-LASIK group were within 0.5 D of target correction and 88% (50/57) eyes within 1.0 D of target correction. 86% (49/57) eyes of FS-LASIK group remained astigmatism ≤ 0.5 D. Detailed visual outcomes were reported in Fig. 1.

### Posterior corneal elevation changes

No statistically significant differences were recorded for PCE, PTE and PME between the SMILE and FS-LASIK groups at baseline (*P* > 0.05).

**Fig. 2:**
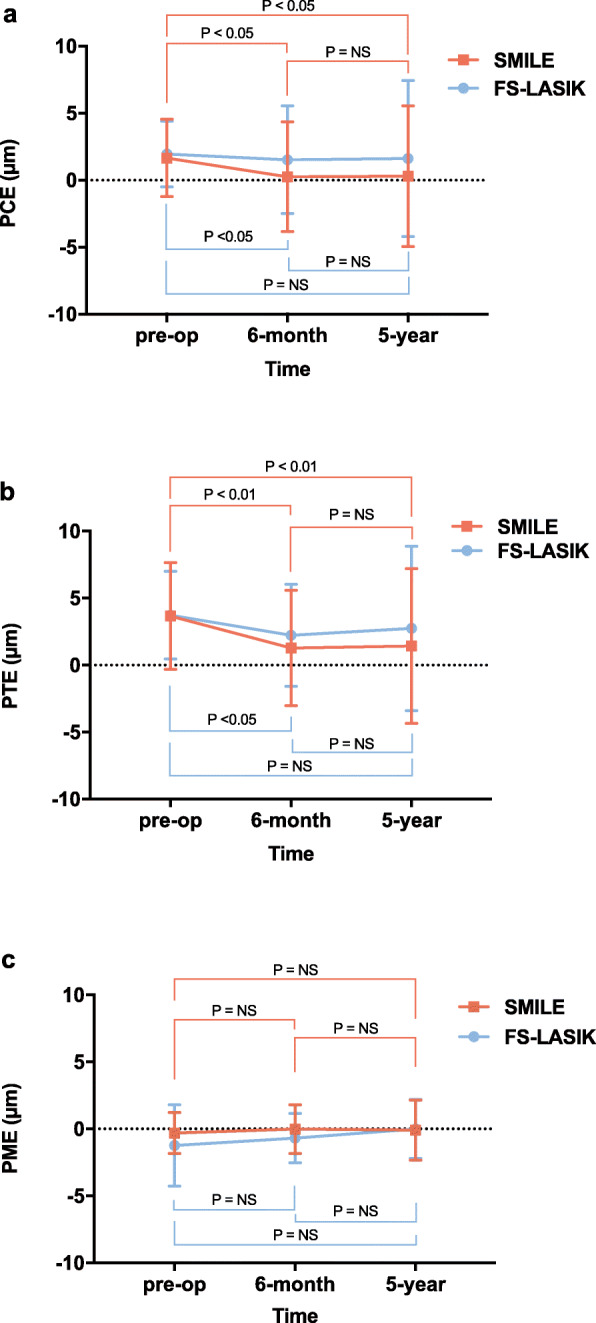
Posterior corneal elevation changes at the 6-month and 5-year follow-up. a. PCE change; b. PTE change; c. PME change. PCE = posterior central elevation; PTE = posterior corneal surface at thinnest point; PME = posterior corneal mean elevation; NS = not significant. *P* values were adjusted for preoperative spherical equivalent in mixed effect model. *P* values less than 0.05 were considered statistically significant

PCE underwent a backward displacement in the 6-month follow-up for both SMILE (dropped by −1.11 ± 2.93 μm, *P* < 0.05) and FS-LASIK groups (dropped by −0.46 ± 3.72 μm, *P* < 0.05) compared with preoperative baseline. PCE in the 6-month follow-up averaged 0.26 ± 4.09 μm for the SMILE group, and 1.53 ± 4.03 μm for the FS-LASIK group. PCE then remained stable in the 5-year follow-up (elevation change: −0.28 ± 4.03 μm, *P* > 0.05) in comparison with 6-month follow-up (Fig. 2a). The five-year postoperative PCE was 0.30 ± 5.25 μm for the SMILE group and 1.63 ± 5.83 μm for the FS-LASIK group.

Pattern of PTE fluctuation was comparable with PCE in chronological order. PTE also underwent a backward displacement in the 6-month follow-up for both SMILE (dropped by −2.04 ± 3.02 μm, *P* < 0.05) and FS-LASIK groups (dropped by −1.28 ± 4.21 μm, *P* < 0.05). Unchanged PTE in 5-year follow-up for SMILE (elevation change: −0.08 ± 4.28 μm, *P* > 0.05) and FS-LASIK (elevation change: −1.42 ± 3.85 μm, *P* > 0.05) groups were recorded compared to 6-month follow-up (Fig. 2b).

PME remained unaffected at 6 months (elevation change: SMILE 0.30 ± 1.54 μm, *P* > 0.05; LASIK 0.29 ± 1.42 μm, *P* > 0.05) and 5 year (elevation change: SMILE 0.32 ± 1.96 μm, *P* > 0.05; LASIK 0.68 ± 2.03 μm, *P* > 0.05) follow-up for both groups compared with preoperative data (Fig. 2c).

No differences of statistical significance were noted between SMILE and FS-LASIK groups in relation to PCE, PTE and PME during each follow-up while adjusting for preoperative SE (*P* > 0.05).

### Associations between posterior corneal stability and ablation ratio

Ablation ratio was strongly correlated with 5-year postoperative PCE (β = 2.68 ± 1.05, *P* < 0.01) and PTE (β = 2.35 ± 1.17, *P* < 0.05) as an independent risk factor while adjusting for age, sex and surgical procedures. Cut-off point for 5-year postoperative raised PCE and PTE was 27.3 and 27.1%, respectively, as determined by maximum Youden’s index. Males (*P* < 0.05) and younger age (*P* < 0.05) were associated with backward displacement of PCE and PTE at the 6-month follow-up. Univariate and multivariate analysis of parameters associated with posterior corneal elevation in each follow-up are summarized in Table 2.

**Table 2:** Parameters associated with posterior corneal elevation at 6-month and 5-year follow-up

Univariate analysis								Multivariate analysis						
**6-month follow-up**		**PCE**		**PTE**		**PME**		**6-month follow-up**	**PCE**		**PTE**		**PME**	
Parameters		Correlation coefficient	*P* value	Correlation coefficient	*P* value	Correlation coefficient	*P* value	Parameters	Correlation coefficient	*P* value	Correlation coefficient	*P* value	Correlation coefficient	*P* value
Sex	Male	−1.32 ± 3.01	< 0.05	−0.48 ± 2.74	< 0.05	−0.24 ± 1.85	0.98	Sex (Male vs. Female)	−1.99 ± 0.74	0.01	−2.15 ± 0.72	0.003	NS	0.84
	Female	1.13 ± 4.23		2.04 ± 4.32		−0.25 ± 1.93								
Age	≥20	−2.00 ± 2.28	0.16	0.80 ± 1.64	0.09	−2.67 ± 3.21	0.13	Age	0.19 ± 0.07	0.003	0.19 ± 0.07	0.01	NS	1.00
	21 ~ 30	0.05 ± 4.28		1.00 ± 4.30		−0.04 ± 1.97								
	31 ~ 40	0.67 ± 3.83		1.30 ± 3.72		−0.39 ± 1.54								
	≥41	3.50 ± 3.42		4.88 ± 3.94		−0.36 ± 1.66								
Surgical group	FS-LASIK	1.34 ± 4.21	0.23	2.09 ± 4.00	0.35	0.03 ± 1.88	0.04	Surgical group (SMILE vs. FS-LASIK)	NS	0.26	NS	0.36	NS	0.42
	SMILE	0.26 ± 4.21		1.23 ± 4.37		−0.82 ± 1.82								
Ablation ratio^a^	High-level	0.69 ± 4.43	0.87	1.69 ± 4.43	0.7	−0.35 ± 1.79	0.63	Ablation ratio	NS	0.76	NS	0.82	NS	0.90
	Low-level	0.55 ± 4.08		1.36 ± 4.14		−0.16 ± 1.98								
**5-year follow-up**		**PCE**		**PTE**		**PME**		**5-year follow-up**	**PCE**		**PTE**		**PME**	
Sex	Male	1.38 ± 4.97	0.6	2.07 ± 5.26	0.95	0.28 ± 2.26	0.38	Gender (Male vs. Female)	NS	0.81	NS	0.90	NS	0.78
	Female	0.76 ± 5.75		1.99 ± 6.20		−0.15 ± 2.25								
Age	≥20	2.43 ± 3.55	0.23	3.57 ± 3.21	0.24	1.23 ± 1.90	< 0.001	Age	NS	0.31	NS	0.28	−0.13 ± 0.04	0.001
	21 ~ 30	1.46 ± 5.23		2.55 ± 5.70		0.44 ± 1.99								
	31 ~ 40	0.13 ± 4.16		1.39 ± 4.79		−0.59 ± 2.24								
	≥41	−1.73 ± 10.27		−1.00 ± 10.42		−2.35 ± 2.38								
Surgical group	FS-LASIK	1.63 ± 5.83	0.18	2.74 ± 6.13	0.22	−0.01 ± 2.21	0.83	Surgical group (SMILE vs. FS-LASIK)	NS	0.75	NS	0.80	NS	0.88
	SMILE	0.30 ± 5.25		1.42 ± 5.78		−0.01 ± 2.27								
Ablation ratio^a^	High-level	2.33 ± 4.68	< 0.05	3.38 ± 4.88	< 0.05	0.20 ± 2.03	0.25	Ablation ratio	2.68 ± 1.05	0.01	2.35 ± 1.17	0.04	NS	0.48
	Low-level	−0.56 ± 5.99		0.61 ± 6.64		−0.28 ± 2.40								

^a^High and low-level were divided by median ablation ratio in univariate analysis. Parameters with P value less than 0.1 in any one of follow-ups were entered into a multivariable analysis using generalized estimating equations. *SMILE=* small incision lenticule extraction; *FS-LASIK*=femtosecond laser-assisted in situ keratomileusis; *PCE*=posterior central elevation; *PTE*=posterior corneal surface at thinnest point; *PME*=posterior corneal mean elevation; *NS*=not significant. *P* values less than 0.05 were considered statistically significant

## Discussion

Pentacam, by manipulating a rotating Scheimpflug camera ﻿to directly construct an elevation map without causing false positive forward elevation ﻿induced by mathematical reconstruction, has now replaced Orbscan II as a standard measurement for corneal topography before and after keratorefractive surgeries [14–17].

With posterior corneal forward displacement proved to be an important warning sign of postoperative corneal instability, posterior corneal elevation has long been adopted in evaluating corneal displacement after nearly all keratorefractive surgeries including PRK [18], laser assisted subepithelial keratomileusis (LASEK), sub-Bowman’s keratomileusis (SBK) [19], LASIK [20] and SMILE [21].

In this study, PCE change (6-month: −1.11 ± 2.93 μm, 5-year: −1.44 ± 3.93 μm) and PTE change (6-month: −2.04 ± 3.02 μm, 5-year: −2.41 ± 4.56 μm) of SMILE group were comparable to Zhao et al. (PCE, 6-month: 0.33 ± 3.94 μm, 3-year: ﻿−1.42 ± 3.48 μm; PTE, 6-month: ﻿1.81 ± 4.08 μm, 3-year: ﻿0.36 ± 4.13 μm) [8] and Zhou et al. (PCE, 6-month: −0.23 ± 2.43 μm, 2-year: −1.18 ± 3.06 μm; PTE, 6-month: ﻿﻿−1.53 ± 3.56 μm, 2-year: ﻿﻿−1.94 ± 3.21 μm) studies [12] with slightly more backward shift in all follow-up data.

A slight backward displacement of PCE and PTE with statistical significance was witnessed at the postoperative 6-month interval in both SMILE and FS-LASIK groups. This phenomenon was observed by Zhou et al. in high myopia patients in their 6-month follow-up after SMILE and FS-LASIK surgeries as well [12]. Yu et al. reported a backward shift of PCE in both SMILE and LASEK group in both 3-month and 3-year follow-ups without recording PTE data [21]. The same short-term backward shift was also noticed in PCE of rabbit eyes post-SMILE surgery [22]. The backward shift of PCE and PTE observed in this study may be explained by wound-remodeling in the early stage [23]. Postoperative thickening of the unablated peripheral stroma could cause flattening of posterior cornea surface [24] and may also contribute to the backward shift of PCE and PTE. Though there were some earlier studies reporting an obvious forward shift after LASIK or LASEK surgeries, most of them described a false positive forward elevation exacerbated by Orbscan [25, 26].

With respect to PME, this study selected posterior elevation of a 4 mm diameter concentric circle averaged for PME calculation whilst some studies picked central 2 or 6 [23, 27] or even 7 mm [28] area for analysis. The lack of protocol in assessing posterior corneal surface has made it difficult to make comparisons between different studies. PME changes of central 4 mm area were of no statistical importance in each follow-up in this study. It could possibly be due to the asphericity of the posterior corneal surface. As discovered by Zhou et al., PME of the temporal area underwent a more obvious backward displacement than nasal, superior and inferior areas [12]. Changes in PME remain unnoticed in our study when 8 points of the concentric 4 mm zone were simply averaged.

In this study, no difference of postoperative posterior corneal elevation change was noted between SMILE and FS-LASIK groups in each follow-up visit after adjusting for preoperative SE. With no adjustment for preoperative data, slightly greater PCE and PME changes could be seen in FS-LASIK group than SMILE group in previous studies [12, 29].

We applied ablation ratio (SMILE: lenticule thickness/CCT; FS-LASIK: ablation depth/CCT), namely percentage of ablated corneal tissue, in this study to evaluate risk factors of postoperative posterior corneal forward elevation instead of adopting “one-size-fits-all” metrics like RBT, ablation depth, lenticule thickness. Ablation ratio was a relatively individualized metric to describe postoperative corneal alteration. Some studies adopted other customized parameters like percent tissue altered (PTA) [30, 31], modified PTA [6] or corneal ratio (corneal depth/pachymetry) [32] to analyze the postoperative biomechanical destabilization. It is worth noting that that the ablation ratio, like any other customized metric, could hardly be a perfect parameter in estimating corneal topographic features based on the fact that the cohesive tensile strength of human cornea stroma is not evenly distributed, but progressively weakened from the anterior to the posterior surface [33].

The ablation ratio was strongly correlated with 5-year postoperative PCE (β = 2.68 ± 1.05, *P* < 0.01) and PTE (β = 2.35 ± 1.17, *P* < 0.05). However, the correlation was not noted in the 6-month follow-up. The missed correlation corroborated with another short-term observation study [34]. Brenner et al. found that in post-LASIK ectasia cases, the ablation ratio had ﻿a strong association with the severity of the ectasia and ablation ratio more than 20% would particularly increase the risk for post-LASIK ectasia CDVA loss [32]. No patients in the SMILE or FS-LASIK groups in this study suffered from iatrogenic keratectasia at the 5-year follow-up. We determined in this study that threshold of ablation ratio to resist a forward displacement of posterior corneal surface was 27.3 and 27.1% for the SMILE and FS-LASIK groups, respectively.

Limitations of this current study include firstly, the study did not adopt randomization after considering patients’ benefits since long-term effect of SMILE surgery remained unknown at the time of enrollment. Secondly, spherical refractive error, cylindrical refractive error and spherical equivalent of two groups were statistically different in this study due to the relative low number of patients and of the non-randomization, which limited the validity of the study. Thirdly, only two follow-up time points were picked in this study with the six-month and five-year follow-up representing a short and long-time observation, respectively. Lastly, this study failed to consider asphericity of the posterior corneal elevation by using BFS instead of best fit toric ellipsoid and simply averaging posterior elevation without differentiating between different hemispheres.

## Conclusions

In summary, postoperative PCE and PTE of SMILE and FS-LASIK groups underwent slight backward displacement at 6 months but remained stable at 5 years follow-up. Ablation ratio was strongly correlated with postoperative posterior corneal elevation. The threshold of the ablation ratio for resisting forward displacement of posterior corneal surface was 27.3 and 27.1% for the SMILE and FS-LASIK groups, respectively.

## Data Availability

Available from the corresponding author upon reasonable request.

## Abbreviations

SMILE: Small incision lenticule extraction
FS-LASIK: Femtosecond laser-assisted in situ keratomileusis
CCT: Central corneal thickness
PCE: Posterior central elevation
PTE: Posterior corneal surface at thinnest point
PME: Posterior corneal mean elevation
PRK: Photorefractive keratectomy
CDVA: Corrected distance visual acuity
RBT: Residual bed thickness
logMAR: Logarithm of the minimal angle of resolution
UDVA: Uncorrected distance visual acuity
D: Diopter
BFS: Best fit sphere
GEE: Generalized estimating equations
LASEK: Laser assisted subepithelial keratomileusis
SBK: Sub-Bowman’s keratomileusis
